# Visceral leishmaniasis during pregnancy: A rare case report from Greece

**DOI:** 10.1371/journal.pntd.0005134

**Published:** 2017-02-16

**Authors:** Periklis Panagopoulos, Vasileios Mitsopoulos, Antonios Papadopoulos, Spyridoula Theodorou, Chrysoula Christodoulaki, Kyriakos Aloupogiannis, Nikolaos Papantoniou

**Affiliations:** 1 Third Department of Obstetrics and Gynaecology, University General Hospital Attikon, Medical School, National and Kapodistrian University of Athens, Athens, Greece; 2 Fourth Department of Internal Medicine, University General Hospital Attikon, Medical School, National and Kapodistrian University of Athens, Athens, Greece; 3 Department of Obstetrics and Gynaecology, Tzaneio General Hospital, Piraeus, Greece; Institute of Tropical Medicine, BELGIUM

## Overview

### Objective

We report here a rare case of visceral leishmaniasis (VL) during pregnancy from Greece that adds to the extremely scarce reports on the topic globally.

### Case report

The patient presented with symptoms and signs of fatigue and cachexia. Physical examination and initial laboratory tests showed afebrile pancytopenia, hypergammaglobulinemia, and hepatosplenomegaly, which was confirmed with an ultrasound scan. Demonstration of amastigote forms of Leishmania parasite on a bone marrow smear confirmed the diagnosis of VL. The patient was treated with Liposomal Amphotericin-B, with excellent results for both the mother and the fetus.

### Conclusion

Although VL is rare in pregnancy, it should always be included in the differential diagnosis in women with compatible symptoms and signs. Treatment of pregnant women is essential in order to reduce the risk of vertical transmission. L-AmB seems to be the drug of choice, based on the high cure rate and the safety profile of the drug.

## Introduction

VL, also known as kala-azar, is an endemic disease in tropical regions, subtropical regions, and the Mediterranean basin, including Greece. The worldwide prevalence of VL is estimated to be approximately 200,000 to 400,000 new cases per year, with more than 20,000 deaths recorded annually [1]. In the Mediterranean European countries, the estimated annual VL incidence is 437 to 639 new cases [2]. VL can be classified as zoonotic or anthroponotic, depending on whether the reservoir host of the parasite is animal or human [3]. The predominant etiological agent globally is *Leishmania donovani* (anthroponotic disease). *L*. *infantum* (synonym *L*. *chagasi*) -derived VL is zoonotic in Europe and the Americas. In Europe and in the Mediterranean countries, *L*. *infantum* is almost exclusively predominant, and the parasites are transmitted by phlebotomine sandflies, with canines being the main reservoir hosts in the urban environments of these countries [4]. The incubation period is usually two to six months but may range from a few weeks to a few years [1]. Clinical manifestations of VL include fever, cachexia, hepatosplenomegaly, anemia, or even pancytopenia, and if untreated, the disease is lethal [5][6]. Although in utero transmission to the fetus occurs rarely, there are scarce reports in literature, and VL infection during pregnancy has been associated with congenital transmission and fetal death [7][8]. The drug of choice for initial treatment in pregnancy is liposomal amphotericin B (L-AmB) because of its safety profile for the mother and the fetus [7].

We present here a case of VL in a pregnant woman from Greece. Informed written consent was obtained prior to any diagnostic procedures or treatment and also prior to publication of case details

## Case report

A 19-year-old pregnant woman of Roma ethnic origin and a permanent resident of an area near Athens with no prior antenatal care and of unknown gestational age presented at the emergency room with fatigue, cachexia, and anorexia. The patient reported that she had two previous uncomplicated pregnancies, with no history of travelling abroad. Physical examination revealed splenomegaly and hypotension with temperature and heart rate within normal ranges. Laboratory investigations revealed mild dysregulation of all three cell lines with a Hct: 21.5%, hemoglobin 6.6 g/dL, leucocyte count 1,880/ml (63.4% neutrophils), platelet count 130,000/μL, and substantial hypoalbuminemia. An abdominal ultrasound revealed hepatosplenomegaly, with liver and spleen longitudinal diameters of 23 cm and 23.5 cm, respectively. A subsequent obstetric ultrasound scan revealed an estimated gestational age of 26+4/40 +/– 14 days, with an estimated fetal weight of 1,095 g and with normal Dopplers of umbilical artery, middle cerebral artery, and ductus venosus.

**Fig 1:**
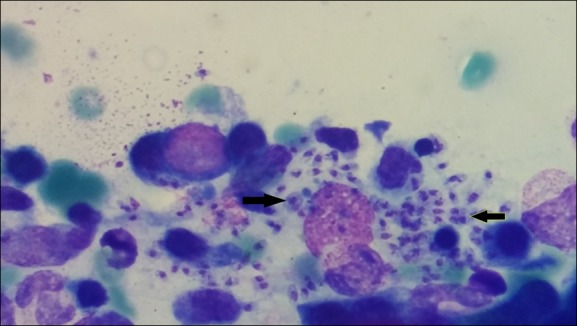
Amastigote forms of *Leishmania* in our patient’s Giemsa-stained bone marrow smear.

Serum electrophoresis was performed before any transfusion and showed hypergammaglobulinemia. She was hospitalized and initially transfused with two units of packed red blood cells (RBCs) and one unit of fresh frozen plasma (FFP); a new full blood count two days later demonstrated a persistent pancytopenia. Two sets of blood cultures and an HIV antibody test were negative. By contrast, the rK39 antigen in immunochromatographic strip format (IT LEISH Rapid Test, Bio-Rad Laboratories) was positive, while IgG/IgM antibodies (ELISA IgG + IgM, Vircell Microbiologists, Granada, Spain) against *Leishmania* were detected. Finally, after thorough counseling, the patient consented to having a bone marrow aspiration performed. A Giemsa-stained smear was prepared, and amastigote forms of *Leishmania* parasite were demonstrated (Fig 1). PCR was impossible because of technical issues, but since the patient was a Greek inhabitant, we assumed that *L*. *infantum* was the most likely pathogen.

L-AmB was started at a dose of 3 mg/kg/day for five days, followed by two more doses on days 14 and 21, according to the United States Food and Drug Administration (FDA) approved regimen that is also used in men and nonpregnant women [9]. The patient’s clinical and laboratory statuses improved beginning on the third day of L-AmB administration. She was discharged from the hospital two days after the administration of the initial five-day treatment. One month after treatment (last dose on day 21), the patient and the fetus were reassessed with new blood tests and ultrasound scans, respectively. Laboratory findings were within normal range, while liver and spleen size were almost normal. The biophysical profile of the fetus was reassuring. Two months after treatment, the patient delivered a healthy term neonate weighing 3,480 g, with an Apgar score of 9 and 10 at first and fifth minute, respectively. The neonate had no signs or symptoms of VL, with laboratory findings within normal range, along with normal abdomen and brain ultrasound scanning. Serologic exams on the neonate during the first days after birth detected IgG/IgM antibodies (ELISA IgG+IgM, as above) against *Leishmania*, along with a positive rK39 antigen in an immunochromatographic strip format (IT LEISH rapid test, as above). The available tests were unable to distinguish IgG from IgM antibodies; the test to detect only IgM antibodies was not offered. Furthermore, a PCR from peripheral blood of the neonate was impossible because of technical issues, and the parents of the neonate did not consent for a bone marrow aspiration on the neonate. The infant became serologically negative within six months of being born, indicating the maternal origin of the antibodies [10][11]. Fourteen months after integration of treatment and 12 months after birth, both our patient and the infant remain completely healthy, with no signs of relapse.

## Discussion

VL is considered endemic in nine European countries, including Greece. In the Greek region, *L*. *infantum* is the responsible species for VL, with competent phlebotomine vectors being *Phlebotomus neglectus*, *P*. *tobbi*, and *P*. *perfiliewi* [1]. During the 2004–2014 period, 606 cases of VL were recorded in Greece [1]. However, infectious diseases are usually under-reported in Greece, thus limiting our knowledge of their actual prevalence. Remarkably, screening control of canines in different regions of the country revealed seropositivity of 20%–24% [1]. Nonetheless, according to our knowledge, this is the second case of VL during pregnancy in Greece [12] and the first that was treated for VL during the antepartum period.

In our case, no apparent mode of infection was recognized. Our patient had never travelled abroad and had never had a blood transfusion, nor had she ever been an intravenous drug user. Nonetheless, the fact that the patient was living near an urban dump bolsters the hypothesis of close contact with *Leishmania*-infected stray dogs and sandflies.

The first congenital case of kala-azar was described in 1926 by Low and Cooke, and up to 2012, there were only 14 cases of congenital transmission of VL reported [7][8][10–13]. Moreover, until 2005, only 18 cases of symptomatic pregnant women treated for VL were reported [7][10]. Until that time in Europe, there were only nine cases of pregnant women suffering from VL that received treatment during pregnancy.

Concerning vertical transmission, it might occur either transplacentally during pregnancy (in utero) or, most likely, during labor via blood exchange from the mother to the child [14][15]. Postnatal transmission, mainly through breastfeeding or transmission of dead parasites, parasite DNA, or their molecules, is excluded [14]. Vertical transmission may be associated with an increased parasite load, immunosuppression, or even genetic predisposition, as is suggested in canine populations [16]. In VL-endemic areas, congenital VL cases cannot be safely distinguished from cases of infection by vector transmission during the first months of life [17]. Moreover, there have been described cases of congenital transmission of VL from asymptomatic or subclinical infected women [15][17].

No established guidelines exist on how to rule out vertical transmission during the first year of life and how to perform the diagnostic follow-up of children during this period. Some experts suggest a histopathologic study of the placenta in order to observe the presence of the parasite [11]. This study was not done in our case, but in most of the congenital VL described in literature, changes in the placenta were typically not observed [8]. In the vast majority of congenitally infected children, the symptoms and signs of VL developed during the first 12 months of life and, in our case, the child remained asymptomatic during that period [15]. If a child is asymptomatic, a serological investigation should be performed during the first year of life [11]. In our case, serologic examination on the neonate, namely the rK39 antigen and the detection of antibodies against *Leishmania* through ELISA, was positive. Unfortunately, the available tests were unable to distinguish IgG from IgM antibodies. The neonate became serologically negative within six months of being born, indicating the maternal origin of the antibodies. However, data available in the literature about the contribution of serologic exams into the surveillance of the neonates are conflicting or at least discouraging [18]. In particular, infants have been described in endemic areas with positive serological markers that never progressed to classical disease, whereas others developed VL while they were serologically negative [18]. Therefore, conventional methods, such as well-characterized serological tests, may be useful, but in high-income settings, they are only screening tools and have limitations on their use [11][18]. A bone marrow aspirate is the gold standard for confirming diagnosis in the newborn [10]. In cases where bone marrow examination is not feasible or negative, a positive PCR result immediately after delivery could indicate the possibility of asymptomatic vertical transmission, but it is not very useful afterwards since it cannot rule out the possibility of vector transmission [18]. In our case, PCR was not possible at that time, and the parents denied the bone marrow examination of the newborn.

During the last decade, Figueiro-Filho et al. reported five new cases of pregnant women in Brazil who were treated for VL, while Mueller et al. and Adam et al. published two relatively large series from Sudan concerning appropriate treatment for VL during pregnancy [11][19][20]. More specifically, Mueller et al. (in a retrospective analysis) reviewed 39 cases of pregnant women suffering from VL who were treated with either sodium stibogluconate, L-AmB, or a combined regimen. The most striking feature was that, in the stibogluconate group, there were 13 (57%) first and second trimester miscarriages during treatment, in contrast to the other two groups [19]. In Adam et al.’s prospective study, among 42 pregnant patients with VL treated with sodium stibogluconate, four died from hepatic encephalopathy during the course of treatment, while two first trimester spontaneous abortions and one congenital infection were also reported [20].

According to WHO, the definition of VL includes a history of fever for two weeks, malaria excluded, along with wasting and either splenomegaly or lymphadenopathy [21][22]. Interestingly enough, in our case, fever was absent, which could mislead and delay the diagnosis of VL, especially in other settings [21]. This is probably explained by the fact that many infected individuals show a subclinical or clinically mild form of the disease and that the number of severe cases with clear clinical manifestations is relatively small [10]. Fever was the main presenting symptom in eight out of the nine pregnant women suffering from VL in Europe until 2005, whereas only one of them was afebrile [23]. Apart from fever, several other symptoms constitute the clinical spectrum of VL, including cachexia, weight loss, splenomegaly, moderate hepatomegaly, lymphadenopathy, and gradually dark discoloration of the skin. Additionally, in advanced stages, hypoalbuminemia may result in edema and ascites, while secondary infections may occur [6]. Furthermore, laboratory findings include anemia and leukopenia in the early stages of the disease and, if untreated, thrombocytopenia, pancytopenia, hypoalbuminemia, marked polyclonal increase in serum immunoglobulins, and elevated levels of transaminases and bilirubin may also occur [6][24]. VL should be differentiated from a series of infectious diseases, such as malaria, brucellosis, typhoid fever, histoplasmosis, schistosomiasis, etc. [10][25] Additionally, leukemia or lymphoma may be suspected because of hematological abnormalities, mainly due to bone marrow suppression, splenomegaly, and hemolysis [25][26].

A wide range of serological and molecular tests contribute to the diagnosis of VL. Among them, rK39 antigen-based immunochromatographic strip format is a useful diagnostic tool with high accuracy rates in the diagnosis of VL in most endemic areas. In particular, in a meta-analysis, the rK39 strip test showed sensitivity and specificity of 98.4%–100% and 81.2%–96.4%, respectively, in specific regions [27]. The rK39-based tests are easy to perform, quick, cheap, and give reproducible results, and can therefore be used for early diagnosis of visceral leishmaniasis [4]. A direct agglutination test (DAT) may also contribute to the diagnosis. In a meta-analysis regarding DAT accuracy, sensitivity and specificity accounted for 94.8% and 85.9%, respectively [27]. Both tests have two main drawbacks. First, they remain positive for many years after treatment, so they cannot be used as a tool for diagnosing relapse or cure [4][27]. Second, both tests are positive in a significant proportion of healthy residential population of endemic areas, mainly due to asymptomatic infections [4][27].

PCR is widely used in developed countries nowadays. It has high sensitivity rates, which might vary, however, depending on the sample tissue used (e.g., blood sample) [6][28]. The predominant drawback of all of these diagnostic tools is that, especially in endemic areas, they are not capable of distinguishing an active disease from an asymptomatic or subclinical infection, in addition to the lack of global standardization or availability [29]. A definite diagnosis requires the demonstration of the parasites in tissue smears of the affected organs (bone marrow, spleen, and, less frequently, lymph nodes) via needle aspiration or biopsy [6][25]. In such cases, PCR is not necessary as a confirmatory test. In our case, the diagnosis was supported by the combination of a positive rK39 antigen in an immunochromatographic strip format test and the positive IgG/IgM antibodies via ELISA. These findings were confirmed by the demonstration of the amastigote forms of *Leishmania* in a Giemsa-stained bone marrow smear, which constitutes the gold standard for the diagnosis of VL [6].

Concerning proper treatment of VL, L-AmB is the drug of choice for the treatment of VL in developed countries and in countries where it can be safely administered with no cost constraints [30]. L-AmB is FDA approved (category B in pregnancy). We used a standard FDA-approved regimen for immunocompetent patients. Different dose regimens have been tested worldwide, including shorter courses with higher daily doses, with good results. Although no published guidelines exist for the use of L-AmB in pregnancy, accumulated data indicate that the drug is highly efficacious and safe compared to other drugs. In one of the largest series (*n* = 23) of pregnant women with VL receiving L-AmB in Eastern Sudan at a total dose of 30 mg/kg for 10 consecutive days, the cure rate was 100%, with no relapses after a six-month follow-up period [31]. In contrast to conventional deoxycholate amphotericin B, L-AmB has the best safety profile, demonstrates fewer and reversible adverse effects to the mother, such as nephrotoxicity and hypokalemia, while no pregnancy loss or maternal death has been observed [8][32]. It should also be stated that no vertical transmission of the disease or any repercussion on the fetus has been reported following the use of L-AmB [32][33]. The main drawback of the drug is its high cost, which explains why pentavalent antimonial compounds are still the drug of choice in most endemic regions of the world, despite longer hospitalization, emergence of resistance, treatment failures, recurrences, and adverse effects in both the fetus and the mother, such as abortions, preterm labors, and stillbirths [20][34]. Moreover, miltefosine is teratogenic in rats and is contraindicated in pregnancy [34].

## Conclusion

Although VL is rare in pregnancy, it should always be included in the differential diagnosis in women with compatible symptoms and signs. A high index of suspicion is required in cases where the clinical picture is atypical and the diagnostic tests may be misleading or incomplete. Treatment of pregnant women is essential to reducing the risk of vertical transmission [12]. L-AmB seems to be the drug of choice, based on the high cure rate and the safety profile of the drug.

### Learning points

- Physicians should have a high index of suspicion for VL in pregnant women with compatible signs and symptoms, but atypical clinical manifestations can mislead or delay the diagnosis
- VL during pregnancy requires treatment to prevent fetal-maternal adverse effects and vertical transmission
- Liposomal Amphotericin-B is the drug of choice due to its efficacy and safety profile
